# Supplementary material for: Arp2/3-dependent endocytosis ensures Cdc42 oscillations by removing Pak1-mediated negative feedback
Source: J Cell Biol. 2024 Jul 16;223(10):e202311139. doi: 10.1083/jcb.202311139 (PMC11259211; doi:10.1083/jcb.202311139)
Supplement: Table S1 — shows parameter values of the wild-type model2, including parameters in model2-1. [file JCB_202311139_TableS1.docx]

**Table S1. Parameter values of the wild-type model2, including parameters in model2-1.**

| **Parameter** | **Description** | **Value**  **(Model-X)** | **Value**  **(Model-Pak1)** | **Unit** | **Range for oscillation search** |
| --- | --- | --- | --- | --- | --- |
| $D_{C}$ | Diffusion of Cdc42-GDP in cytoplasm | 3 | 3 | a.u.  length/s | (0,10) |
| $D_{S}$ | Diffusion of Scd1 in cytoplasm | 3 | 3 | a.u.  length/s | (0,10) |
| $D_{P}$ | Diffusion of Pak1 in cytoplasm | 3 | 3 | a.u.  length/s | (0,10) |
| $k_{c}$ | Activation rate constant of Cdc42 at tips | 1.5 | 1.7 |  | (0,2) |
| $n_{sc}$ | Cooperativity of active Cdc42 self-regulation | 4.5 | 5 |  | (0,6) |
| $K_{sc}$ | Threshold of Cdc42 activation by Scd1 | 0.545 | 0.5 | a.u | (0,1) |
| $\delta_{c}$ | Detachment rate constant of active Cdc42 | 0.3 | 0.3 | /min | (0,1) |
| $k_{s}$ | Accumulation rate constant of Scd1 at tips | 1.5 | 1.5 |  | (0,2) |
| $n_{cs}$ | Cooperativity of Scd1 by active Cdc42 | 1 | 1 |  | (0,6) |
| $K_{cs}$ | Threshold of Scd1 accumulation by active Cdc42 | 1 | 1 | a.u. | (0,2) |
| $K_{ps}$ | Threshold of Scd1 inhibition by Pak1 | 0.085 | 0.1 | a.u. | (0,1) |
| $n_{ps}$ | Cooperativity of Scd1 by Pak1 | 3 | 3 |  | (0,6) |
| $\delta_{s}$ | Detachment rate constant of Scd1 | 0.1 | 0.1 | /min | (0,1) |
| $k_{p}$ | Attachment rate constant of Pak1 at tips | 2 | 1 |  | (0,2) |
| $n_{cp}$ | Cooperativity of Pak1 by active Cdc42 | 3 | 3 |  | (0,6) |
| $K_{cp}$ | Threshold of Pak1 attachment by active Cdc42 | 0.5 | 1 | a.u. | (0,2) |
| $\delta_{p}$ | Detachment rate constant of Pak1 | 0.4 | 0.3 | /min | (0,2) |
| a1 | Strength constant of second negative signaling pathway |  | 0.5 |  | (0,1) |
| $n_{pe}$ | Cooperativity of endocytosis/patches by Pak1 |  | 3 |  | (0,6) |
| $K_{pe}$ | Threshold of endocytosis by Pak1 |  | 0.3 | a.u. | (0,2) |
| $k_{o}$ | Cooperativity of endocytosis/patches by other elements |  | 0.2 |  | (0,1) |

* a.u. is an arbitrary unit of concentration.

** a.u. length is an arbitrary unit of length.

Modified parameters for mutants and Model3 were labeled above their corresponding figures.
