# Supplementary material for: Arp2/3-dependent endocytosis ensures Cdc42 oscillations by removing Pak1-mediated negative feedback
Source: J Cell Biol. 2024 Jul 16;223(10):e202311139. doi: 10.1083/jcb.202311139 (PMC11259211; doi:10.1083/jcb.202311139)
Supplement: Table S2 — shows parameter sets comparisons. [file JCB_202311139_TableS2.docx]

**Table S2. Parameter sets comparisons.**

| **Parameter** | **Value 1**  **(Model-Pak1)** | **Value 2**  **(Model-Pak1)** | **Value Change % vs. Value1** | **Value 3**  **(Model-Pak1)** | **Value Change % vs. Value1** | **Unit** | **Range for oscillation search** |
| --- | --- | --- | --- | --- | --- | --- | --- |
| $D_{C}$ | 3 | 2 | 10% | 5 | 20% | a.u.  length/s | (0,10) |
| $D_{S}$ | 3 | 2 | 10% | 5 | 20% | a.u.  length/s | (0,10) |
| $D_{P}$ | 3 | 2 | 10% | 5 | 20% | a.u.  length/s | (0,10) |
| $k_{c}$ | 1.7 | 1 | 35% | 2 | 15% |  | (0,2) |
| $n_{sc}$ | 5 | 2 | 50% | 3 | 33% |  | (0,6) |
| $K_{sc}$ | 0.5 | 1 | 50% | 0.1 | 40% | a.u | (0,1) |
| $\delta_{c}$ | 0.3 | 0.07 | 23% | 0.5 | 20% | /min | (0,1) |
| $k_{s}$ | 1.5 | 1 | 25% | 2 | 25% |  | (0,2) |
| $n_{cs}$ | 1 | 2 | 16% | 3 | 33% |  | (0,6) |
| $K_{cs}$ | 1 | 0.5 | 25% | 0.6 | 20% | a.u. | (0,2) |
| $K_{ps}$ | 0.1 | 0.2 | 10% | 0.4 | 30% | a.u. | (0,1) |
| $n_{ps}$ | 3 | 5 | 33% | 6 | 50% |  | (0,6) |
| $\delta_{s}$ | 0.1 | 0.07 | 3% | 0.3 | 20% | /min | (0,1) |
| $k_{p}$ | 1 | 0.4 | 30% | 2 | 50% |  | (0,2) |
| $n_{cp}$ | 3 | 5 | 33% | 4 | 16% |  | (0,6) |
| $K_{cp}$ | 1 | 0.6 | 20% | 0.8 | 10% | a.u. | (0,2) |
| $\delta_{p}$ | 0.3 | 0.1 | 10% | 1 | 35% | /min | (0,2) |
| a1 | 0.5 | 0.5 | 0 | 0.1 | 40% |  | (0,1) |
| $n_{pe}$ | 3 | 2 | 16% | 5 | 33% |  | (0,6) |
| $K_{pe}$ | 0.3 | 0.6 | 15% | 1.3 | 50% | a.u. | (0,2) |
| $k_{o}$ | 0.2 | 0.2 | 0 | 0.4 | 20% |  | (0,1) |

* a.u. is an arbitrary unit of concentration.

** a.u. length is an arbitrary unit of length.
