# Supplementary material for: Arp2/3-dependent endocytosis ensures Cdc42 oscillations by removing Pak1-mediated negative feedback
Source: J Cell Biol. 2024 Jul 16;223(10):e202311139. doi: 10.1083/jcb.202311139 (PMC11259211; doi:10.1083/jcb.202311139)
Supplement: Table S3 — shows strain list. [file JCB_202311139_TableS3.docx]

| **Strain** | **Genotype** | **Origin** |
| --- | --- | --- |
| PN567 | *h+ ade6-704 leu1-32 ura4-d18* | P. Nurse |
| FV 781 | *h+ Rga4-GFP: KanMx* | F. Verde |
| FV1335 | *h+ CRIB-3xGFP: ura4+* | F. Verde |
| YMD 322 | *CRIB-3xGFP: ura+ Scd2-mCherry: NatMX* | Das Lab |
| YMD 808 | *for3∆::KanMx CRIB-3xGFP: ura+* | This Study |
| YMD 1429 | *for3∆::KanMx Gef1-mNG: KanMx* | This Study |
| YMD 1441 | *for3∆::KanMx Scd1-mNG: KanMx* | This Study |
| YMD 1762 | *Pak1-mEGFP: KanMx Scd1-tdTomato: KanMx* | This Study (JM 4782 Gift from J. Mosley) |
| YMD 1764 | *h+ Pak1-mEGFP: KanMx* | This Study (JM4782 Gift from J. Mosley) |
| YMD 1766 | *for3∆::KanMx Scd1-mNG: KanMx* | This Study |
| YMD 1784 | *orb2-34::ura+ Scd1-mNG: KanMx* | This Study |
| YMD 1820 | *CRIB-mCherry: ade-, leu-, ura- Pak1-mEGFP: KanMx* | This Study |
| YMD 1938 | *orb2-34::ura+ fim1-mCherry:* *NatMX* | This Study (VS888-3 Gift from V. Sirotkin) |
| YMD 2092 | *fim1-mCherry:* *NatMX Pak1-mEGFP: KanMx* | This Study |
| YMD 2043 | *myo1Δ::KanMX Pak1-mEGFP: KanMx* | This Study |
| YMD 2160 | *myo1Δ::KanMX CRIB-3xGFP: ura+* | This Study |
| YMD 2191 | *CRIB-3xGFP: ura+ Scd1-tdTomato: KanMX* | This Study |
| YMD 2232 | *myo1Δ::KanMX Scd1-mNG:KanMX* | This Study |
| YMD 2233 | *Scd1-tdTomato: KanMX CRIB-3xGFP: ura+* | This Study |
| YMD 2266 | *rga4Δ::* ura+ *rga6Δ Pak1-mEGFP:KanMx* | This Study |
| YMD 2267 | *gef1Δ::ura+ Pak1-mEGFP:KanMx* | This Study |
| YMD 2268 | *Pak1-mEGFP: KanMx Scd2-mCherry: NatMX* | This Study |
| YMD 2269 | *scd1Δ::ura+ Pak1-mEGFP: KanMx* | This Study |

**Table S3. Strain list**
